# Managing lifestyle change to reduce coronary risk: a synthesis of qualitative research on peoples’ experiences

**DOI:** 10.1186/1471-2261-14-96

**Published:** 2014-08-05

**Authors:** Felicity Astin, Judith Horrocks, S Jose Closs

**Affiliations:** 1 School of Nursing, Midwifery and Social Work, Mary Seacole Building, Salford, UK; 2 School of Healthcare, Baines Wing, University of Leeds, Leeds LS2 9UT, UK

## Abstract

**Background:**

Coronary heart disease is an incurable condition. The only approach known to slow its progression is healthy lifestyle change and concordance with cardio-protective medicines. Few people fully succeed in these daily activities so potential health improvements are not fully realised. Little is known about peoples’ experiences of managing lifestyle change. The aim of this study was to synthesise qualitative research to explain how participants make lifestyle change after a cardiac event and explore this within the wider illness experience.

**Methods:**

A qualitative synthesis was conducted drawing upon the principles of meta-ethnography. Qualitative studies were identified through a systematic search of 7 databases using explicit criteria. Key concepts were identified and translated across studies. Findings were discussed and diagrammed during a series of audiotaped meetings.

**Results:**

The final synthesis is grounded in findings from 27 studies, with over 500 participants (56% male) across 8 countries. All participants experienced a change in their self-identity from what was ‘familiar’ to ‘unfamiliar’. The transition process involved ‘finding new limits and a life worth living’ , ‘finding support for self’ and ‘finding a new normal’. Analyses of these concepts led to the generation of a third order construct, namely an ongoing process of ‘reassessing past, present and future lives’ as participants considered their changed identity. Participants experienced a strong urge to get back to ‘normal’. Support from family and friends could enable or constrain life change and lifestyle changes. Lifestyle change was but one small part of a wider ‘life’ change that occurred.

**Conclusions:**

The final synthesis presents an interpretation, not evident in the primary studies, of a person-centred model to explain how lifestyle change is situated within ‘wider’ life changes. The magnitude of individual responses to a changed health status varied. Participants experienced distress as their notion of self identity shifted and emotions that reflected the various stages of the grief process were evident in participants’ accounts. The process of self-managing lifestyle took place through experiential learning; the level of engagement with lifestyle change reflected an individual’s unique view of the balance needed to manage ‘realistic change’ whilst leading to a life that was perceived as ‘worth living’. Findings highlight the importance of providing person centred care that aligns with both psychological and physical dimensions of recovery which are inextricably linked.

## Background

The global epidemic of non-communicable diseases (NCDs), such as cardiovascular disease (CVD), represents a major public health challenge and accounts for over half of the global disease burden [1]. Although CVD mortality has decreased in many European countries it nevertheless accounts for almost half of all deaths [2]. Global action plans have been developed to address the burden of NCDs with initiatives aimed at supporting people to self-manage their health [3]. Coronary heart disease (CHD) is one manifestation of CVD and has no known cure. Prevention initiatives, such as the adoption of healthy lifestyle habits and adherence to prescribed medications, offer an evidence based approach, proven to reduce cardiac mortality and morbidity [4]. Despite the compelling evidence about the health benefits of making lifestyle changes for people with CHD, recommendations have not fully translated into improved clinical outcomes as only 50% of individuals adhere to such recommendations [5]. Moreover international surveys have shown that less than 50% of people who are eligible for cardiac rehabilitation, an effective secondary prevention intervention, actually attend [6].

Different academic disciplines have used different ‘lenses’ to examine factors that influence an individual’s ability, or opportunity, to make lifestyle changes to maintain or improve health. Social and behavioral science theories have been used to explain health behavior and inform the development of behaviour change interventions such as the transtheoretical model of change [7], the health belief model [8], social cognitive theory [9] and illness representations as part of self-regulation theory [10]. In this study we have chosen to adopt a person centred approach to better understand patients’ experiences of making lifestyle change from their own unique perspective [11]. Our synthesis focuses specifically upon influences at the level of the individual, their disease and illness experience.

Myriad published qualitative studies have provided detailed accounts of peoples’ recovery experiences following a cardiac event [12,13] but few have focused upon the process of making lifestyle changes to reduce coronary risk. Self-management, a term derived from health policy, describes a person’s ability, to live with and manage a NCD on a day-to-day basis. This may include the management of symptoms, medicines, lifestyle changes and associated physical, functional, emotional, cognitive and social limitations.

Whilst individuals have always played a central role in managing their own health, support from social and professional networks is, without doubt, a valuable adjunct [14]. The World Health Organization states that health professionals should be equipped with the competencies to provide self-management support [15]. To date the notion of self-management support has generally focused upon the transference of knowledge and skills from health professionals, to inform and enable patients, through a process of education. Less attention has been focused upon the process of making lifestyle change from the participant’s perspective and how this links to the broader illness experience. With World Health Organization policy emphasis firmly fixed upon the provision of people-centred care [16] we would suggest this as a significant deficiency particularly as the role of the health professional is to provide self-management support. There is a pressing need to better understand how participant’s engage with secondary prevention behaviours after a cardiac event, such as making lifestyle changes and managing medications which reduces mortality. The aim of this study was to conduct a synthesis of qualitative data to explain how participants made lifestyle changes after a cardiac event and connect this to the illness experience within which this activity is situated. To our knowledge this is the first qualitative synthesis that has addressed this important topic using a meta-ethnographic approach.

## Methods

### Study design

While there are well accepted methodological approaches to guide researchers in the synthesis of quantitative data [17], the same is not true for methods to collate qualitative evidence [18]. There are several approaches that can be used to amalgamate findings from individual qualitative studies to create a new, higher level understanding of a phenomenon [19]. The choice of strategy used to synthesise qualitative research evidence tends to be informed by the world views of those involved in the analytical process. An integrative approach to evidence synthesis tends to emphasise the aggregation of data whereas an interpretative approach focuses upon the development of concepts; the latter approach characterises meta-ethnography which aims to understand how concepts connect across studies to explain a specific phenomenon [20]. As most qualitative methodology is supported by an interpretative paradigm we chose to adopt the interpretative approach exemplified in meta-ethnography [20], an approach originally used as a way of drawing together information from ethnographic studies and subsequently adapted as a method of synthesising data derived from a wider range of qualitative approaches [21-23]. No consensus exists about the most appropriate sampling strategy for meta-ethnographic studies. Our aim was to sample relevant studies that reported concepts that could make a meaningful contribution our synthesis. We used a theoretical approach to include studies from a range of international settings which represented the experiences of both men and women.

### Search strategy methods

Conducting literature searches for qualitative studies is problematic as there is no single recognised approach that is comprehensive. The use of simple search terms is as effective as more complex approaches [24]. We chose to use three approaches to identify relevant papers for our synthesis after seeking advice from an expert Information Technologist. These were a database searches, papers identified through personal knowledge and ‘snowballing’ using references from key papers to identify others [25]. We conducted scoping exercises to develop a robust search strategy. We started with a broad approach using three concepts; ‘self-care’ (self care or self-care) as either a MESH or keyword, keywords linked to the accepted terms used to describe cardiac conditions in Cochrane reviews e.g. ‘myocardial infarct$’ or ‘angioplasty’ , and third, terms that would identify qualitative research e.g. ‘interview’ , ‘qualitative’. This approach was piloted but did not identify some key studies already known to the authors. A second parallel search was run using “lifestyle” as a MESH or keyword combined with the aforementioned cardiac and qualitative search terms. This approach identified a broader range of relevant papers. Searches were run in 2011 in multiple databases (Medline, Embase Classic and Embase, PsycInfo, Cinahl EBSCO, Web of Science, Cochrane and ASSIA) and limited to dates 1990-2011. A comprehensive search of grey literature was not conducted. Inclusion and exclusion criteria are shown below:

a) Study population: people diagnosed with ischaemic heart disease who have experienced a prior ‘cardiac event’ which could be initial diagnosis, Percutaneous Coronary Intervention or Acute Myocardial Infarction (Participants with a diagnosis of Heart Failure were excluded as they experience more marked symptoms which are known to influence self-management activities).

b) Study Aim: the study aimed to report patients’ experiences of making lifestyle changes to reduce coronary risk and provided substantive data on this topic.

c) Methodology of Included Studies: the study must report primary research with qualitative findings either singly, or in combination, with other methods (e.g. quantitative methods).

d) Language: The full paper must be available in English language.

### Data analysis

Following the completion of the search extracted papers were independently assessed against selection criteria by at least 2 researchers. The included papers were then appraised using the CASP framework [26]. This approach was used to facilitate discussion rather than to exclude papers as we chose to adopt the methodological stance which was to include any paper that could contribute something to the synthesis [27]. Data were extracted (JH) and tabulated to provide a summary of studies. A second researcher (FA) checked the extracted data for accuracy. Each paper was then read and reread by three researchers (FA, JH, SJC). In the same way as a primary study may progress from descriptive to an explanatory analysis [28], the researchers engaged in a process of translating concepts across studies. Each study was considered to be an independent ‘piece’ of a larger explanatory jigsaw that would contribute to a new interpretation, not evident in the primary studies, but nevertheless grounded in the corpus of data. There is no consensus about the order in which included studies should be analysed in meta-ethnography. We chose to adopt a pragmatic approach and selected a published study [29] that described a comprehensive ethnographic study of people recovering from acute myocardial infarction (AMI). This provided us with what we considered to be a high quality ‘index’ account characterised by in-depth descriptions from which we could identify preliminary themes and categories. These findings were compared and contrasted across each of the other 26 studies in an iterative cyclical process, similar to the process of constant comparison that exemplifies grounded theory [30], supported by a series of team meetings. We considered how key concepts might relate across studies and identified where there were differences. Our aim was to identify concepts that were similar, divergent, or inconsistent across studies, from which to develop a line of argument to explain patients’ experiences of making lifestyle changes after a cardiac event. Importantly we wanted to understand how lifestyle change is situated within the broader illness experience to better understand how health professionals can give self-management support.

A number of approaches were adopted to support study rigour. Researchers read the included studies independently and identified key themes and concepts within each study. A series of meetings was convened and during each meeting 2-4 studies were discussed in detail. Consensus was reached about the significance, coherence and explanatory power of key concepts, how concepts translated across studies and agreement about an appropriate point to cease sampling when concepts were established and confirmed. Researchers also engaged in a reflexive process that informed discussions about how their own experiences could potentially bias interpretations. Discussions were audio taped and memos recorded to inform the analytical process.

## Results

**Figure 1:**
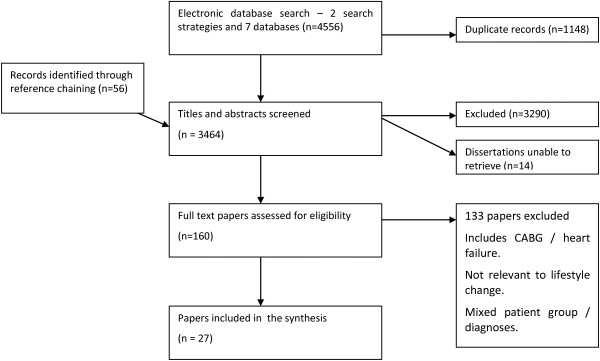
Flow chart of selection of papers.

Figure 1 shows a flow chart of study selection and Table 1 a summary of the extracted data. The final sample represented data from 27 studies with over 500 participants (56% male) representing eight countries. Most studies were conducted in England or Canada with the remainder from other European countries, USA or Australia. All participants were diagnosed with ischaemic heart disease and the majority had suffered an AMI. Participants were interviewed either following diagnosis or 2 days to 25 years after AMI.

**Table 1:** Characteristics of included studies

**Author and country of origin**	**Aim of study, design, method**	**Cohort – detail if stated**	**Main findings**
1. Boutin-Foster (2005) USA [31]	To evaluate the types of instrumental social support that people with CAD find helpful to health behaviour modification.	63 participants (Mean age 69 years. 60% male.	The types of instrumental support perceived as most helpful were those that made it easier to engage in healthy behaviours alleviated stressful situations and facilitated the process of receiving medical care.
	Phenomenological interview study using semi-structured questionnaires.	Diagnosed with coronary artery disease and recruited from a coronary care unit in a single centre.	
2. Condon & McCarthy (2006) Ireland [32]	To explore patients’ perspectives of making lifestyle changes post MI.	10 participants (38–75 years of age) 90% male.	Four main themes: lifestyle warning signs, taking responsibility for lifestyle changes, professional support and looking forward to the future.
	Qualitative descriptive interview study.	All post AMI. Community setting. Interviewed 6 weeks post discharge. All had completed Phase 1 cardiac rehabilitation.	
			Findings highlight the difficulty of making too many lifestyle changes at once as well as a lack of professional support.
			Overprotection from family members creates frustration and aggravation for participants.
3. Coyle (2009) USA [33]	To determine what patients thought was important after experiencing an acute MI and performing self-care.	62 participants (Mean age 63 years, range 37–86 years), 63% male (25% African American).	Seven main themes: The story surrounding the MI, symptom explanations, stresses and loss surfaced during the reflections, fearing death, changing self-care behaviours, reflections on death and gratefulness.
	Part of larger quantitative study – one question asked by telephone.	All post AMI and recruited from a single centre with follow-up at 2 weeks and 30 days post hospital discharge.	
	Colaizzi method of phenomenological analysis.		
4. Doiron-Maillet & Meagher-Stewart (2003) Canada [34]	To explore younger women’s perceptions of their process of recovery following an MI.	8 participants (33–61 years of age), 100% female.	The main themes were: Living with uncertainty, truths learned from others, rude awakening, disconnected knowing and reconnecting self. Women with heart disease were living with an overwhelming sense of uncertainty.
	Interview study using interpretive enquiry with feminist perspective.	Interviewed within 2 weeks of discharge, post AMI and 6–8 weeks later.	
5. Eastwood (2001) Australia [35]	To provide information on change of risk factor behaviour after a PTCA/stent.	4 participants. (100% male), no age specified.	The major influences on whether or not to adopt behaviour change are a new positive health perspective, family considerations, return to work issues and a reluctance to participate in cardiac rehabilitation issues.
	Interview study using a descriptive naturalistic approach.	All were 3 months post PTCA/stent.	
6. Gambling (2003) UK [36]	To investigate understanding of the factors perceived as important in reducing CHD risk factors.	30 to 40 participants. 32–85 years of age.	Main themes were: coping with the diagnosis, participants’ perception of the aetiology of CHD, information seeking behaviour, lifestyle changes, and participants’ appraisal of risk factors relating to CHD.
	Focus group study.	All were post AMI or diagnosed with angina. Recruited from a self-help group, 6 months - 2 years since diagnosis	
			Patients have difficulty processing health information and needed individualised information to help them understand their own risk factors and the necessary action to take.
7. Gregory et al. (2005) Working Paper Scotland [37]	To identify patients’ views and experiences of recovery and rehabilitation from CHD.	53 participants (Aged <65 years), 66% male.	Main themes were: advice and help making sense of the experience and relevance of lifestyle changes, putting advice into practice, barriers to leading a normal life and how things could be improved.
	Focus groups and interviews and interviews analysed using principles drawn from grounded theory.	All were post AMI in a community setting. 2-3 yrs post discharge	
8. Gregory et al. (2005) Scotland [29]	To identify barriers to and facilitators of following advice about lifestyle change and maintenance after an MI.	53 participants (Aged <65 years) 66% male.	A major finding was participants’ desires for long-term monitoring and support which would include regular contact with health professionals, help in following lifestyle advice, reassurance and shared experiences with other MI survivors.
	Focus groups and interviews analysed using principles drawn from grounded theory.	All were post AMI in a community setting. 2-3 yrs post discharge	
9. Gulanick et al. (1998) USA [38]	To examine patients’ responses to suggested lifestyle changes after their PTCA procedure, to identify barriers and facilitators to risk factor reduction, to identify sources of health information, and to elicit suggestions for nursing interventions to aid in long-term recovery.	55 participants, post PTCA*, (Mean age 61 years, range 34–74 years), 47% male.	The main facilitators to lifestyle change were: Permission to cheat, wanting to stay alive, belief that had some control by reducing risk factors, peer support found in cardiac rehabilitation, stress reduction through relaxation or yoga.
		Community setting with some having attended cardiac rehabilitation, 3-18 months post PTCA.	
	A focus group study informed by Cox’s interaction model of client health behaviour framework. Analysis conducted using principles of grounded theory.		The barriers to lifestyle change were: Lack of spousal or family support, powerless to alter disease progression, lack of willpower, fear of overexertion, difficulty coping with stress.
10. Jensen & Petersson (2003) Denmark [39]	To investigate patients’ experiences of illness after a first MI, focusing on life situation and the recovery process over time.	30 participants (40–89 years of age) 73% male.	A core finding was the uncertainty of the life situation with four categories: treatment seeking behaviour, existential threat, preventing another coronary and a need for knowledge and support.
	Semi-structured interviews based on Lunde’s perception model.	Post AMI and interviewed twice: 2nd/3rd day on ward and 17wks post-admission.	
11. Johansson et al. (2003) Sweden [40]	To explore women’s’ experiences after an MI.	8 participants (No ages provided), 100% female.	Women’s’ experience is characterised by uncertainty and a loss of context. Their relationship with their body and the lived world is interrupted and well-being is regained through reconciliation with their body and illness.
	Interviews using a phenomenological approach.	Contacted through patient association. Interviewed 2–25 years post AMI.	
12. Johnson & Morse (1990) Canada [41]	To examine the process of adjustment that individuals experience after an MI.	14 participants (43–72 years of age), 50% male.	Four stages of adjustment are identified: defending oneself, coming to terms, learning to live and living again. A core theme to all stages is the importance of control; the perceived loss of control and the struggle to regain control.
	Unstructured interviews using a grounded theory approach.	Selected from cardiac rehabilitation and cardiac self-help group. All 1–45 months post AMI.	
13. Kerr & Fothergill-Bourbonnais (2002) Canada [42]	To examine the experience of recovery in women aged 65+ during initial recovery from an MI.	7 participants (Mean age 74 years, range 67–86 years of age), 100% female.	Four main themes were: life is scattered, trying to make sense of it, learning to live with it and getting settled.
	Unstructured interactive interviews using a Heideggerian phenomenological approach.	All post AMI and interviewed within 5 weeks of discharge.	Recovery was likened to a “mosaic” where women had to create a new picture for themselves.
14. MacInnes (2005) England [43]	To explore relationships between illness perceptions and adoption of health-promoting behaviours and attendance at cardiac rehabilitation for women after an acute MI.	10 participants (Mean age 72 years), 100% female.	Main themes were: stress as a cause of illness, loss of confidence and inability to complete household chores, the episodic nature of the illness and a perceived lack of control.
		All referred to cardiac rehabilitation and interviewed 3 months post MI.	
	Interview study using Leventhal and Nerenz’s self-regulatory model of illness behaviour as a theoretical framework		
15. McGillion (2007) Canada [44]	To examine potential shifts in the meaning of cardiac pain after a 6 week angina psychoeducational programme. Interview study with descriptive analysis.	Subsample of 66 participants drawn from a larger sample (130) recruited for an RCT.	Angina pain changes from being a burdensome life change to a pain problem requiring self-management in order to preserve life goals and functioning.
		(Mean age 67 years), 80% male, 73% Caucasian).	
		All had chronic stable angina and ischaemic heart disease and an average of 6 years living with angina. Attending a psychoeducational programme for chronic angina self-management.	
16. Mohan, Wilkes & Jackson (2008) Australia [45]	To report lifestyle factors of Asian Indians in Australia in relation to coronary heart disease.	8 participants (Aged 31–80 years, 63% male. Asian Indians).	The main themes were: diet, social and religious customs, exercise, stress, help-seeking behaviour, impact of migration.
	Semi-structured in-depth interviews using a naturalistic approach.	All post AMI in a community setting and interviewed at least 6 months since AMI.	Knowledge of risk factors did not help participants to follow a healthy lifestyle. Any changes made lacked consistency and continuity.
17. Ononeze (2009) Ireland [46]	To explore the individual experience of heart disease and the implication in heart disease prevention and management in the West of Ireland.	26 participants (Mean age 68 years, 62% male).	The main themes were: making sense and coming to terms with illness, increased understanding and learning to live with illness, and managing everyday life with illness.
	In-depth interviews study using a grounded theory approach.	Post AMI or angina from community setting with mixture of paid for and free healthcare. 1.5 - 21 years living with heart disease.	
18. Paquet et al. (2005) Canada [47]	To describe how cardiac patients experience the first 3 months following a cardiac event requiring hospitalization.	20 participants (Mean age 69.6 years, range 50–91 years), 80% male.	The main themes were categorised into: elements at the personal level, elements at the environmental level and elements in interaction at the personal and the environmental level.
		Post AMI, angina or PCA requiring hospitalization, community setting and interviewed 3 months after hospitalization.	
	Focus group study		
			Participants focused on stress management rather than on modifying health habits.
19. Roebuck et al. (2001) England [48]	To explore and gain insights into the effects of myocardial infarction on health-related quality of life.	31 participants (28–74 years of age), 66% male.	The main themes were: physical activity/symptoms, insecurity, emotional reactions, dependency, lifestyle modification, concerns over medication.
	Semi-structured interviews	Interviewed post AMI, in community setting 6 weeks after discharge but prior to cardiac rehabilitation.	
			Breathlessness, insecurity and feelings of overprotection were major problems and participants were dissatisfied with the provision of information and support.
20. Sjostrom-Strand (2006) Sweden [49]	To describe and explore how women cope with stress at the time of and after an MI.	20 participants (30–80 years of age), 100% female.	Cogitative actions, social belonging and emotional diversion were identified as ways of managing stress at the time of and after the MI.
	A descriptive interview study using a phenomenographic approach.	Interviewed in hospital setting (Day 2–3 post AMI) and then between 4–10 months later in community setting.	
21. Sutherland & Jensen (2000) Canada [50]	To explore and describe elderly (70+ years) women’s perceptions of having an MI.	11 participants (70–85 years of age), 100% female.	The main themes were: searching for a diagnosis, being hit with the reality, moving with the change, discovering the nature of the change, adjusting to the change.
	Interview study	Interviewed in a community setting 8 weeks post AMI.	
			Women are met with the constant challenge of being in control, managing uncertainty, making sense, being independent and sheltering others.
22. Warren-Findlow & Prohaska (2008) USA [51]	To examine how family members support or inhibit African American women’s efforts to manage their health conditions.	12 participants (50–73 years of age), 100% female. African - American women only, from a low income minority population.	Most women lived in multi-generational households. Instrumental support was given by family members. Informational support was based on family history of heart disease and behavioural support either reinforced or discouraged lifestyle behaviour changes.
	27 in-depth interviews over a 2 year period. Data analysed using grounded theory.	All had stable CAD.	
23. Warren-Findlow & Issel (2010) USA [52]	To examine stress and coping in older African-American women with CHD.	12 participants. Mean age 62 years (50–73 years of age). 100% female. African - American women only.	Stress was perceived as a cause of CHD. Women used their own family history of heart disease as a reference by which to evaluate their own health. Emotional coping, such as “not worrying” and cognitive coping in the form of spiritual beliefs were used by women.
	Multiple in-depth interviews over a 2 year period.	All had CAD.	
24. White et al. (2010) UK [53]	To explore medicine taking and lifestyle changes in patients after a cardiac rehabilitation programme.	15 participants (42–72 years of age), 73% male.	Participants had unmet informational needs about lifestyle change. They wanted more individualised information and advice. Participants tended to only maintain lifestyle changes that they perceived as causes of their heart attack, but perceived causes could change over time.
	In-depth interviews.	All post AMI and cardiac rehabilitation. Interviewed 3 and 12 months post cardiac rehabilitation.	
25. White et al. (201 1) UK [54]	To explore cardiac rehabilitation patients perspectives on making and maintaining dietary change.	15 participants (42–72 years of age), 73% male.	Participants only made and maintained dietary changes if they perceived their diet to be a cause of their CHD. Dietary changes involved “cutting things out” and no changes were made if they felt that they didn’t need to cut things out.
	In depth interviews.	All post AMI and cardiac rehabilitation. Interviewed 3 and 12 months post cardiac rehabilitation.	
26. Wiles (1998) England [55]	To examine the understandings and beliefs about heart attack and recovery and how lifestyle change fits into these understandings.	25 participants (34–80 years of age), 52% male.	As people recover from the shock of heart attack they begin to lose trust in health professionals accounts of cause and recovery and perceive lifestyle change as an action that cannot guarantee protection from a further heart attack.
	In-depth interview study.	Recruited from a larger RCT study sample. All post AMI and interviewed at 2 weeks and 5 months after hospital discharge.	
27. Wiles & Kinmonth (2001) England [56]	To explore patients’ understandings of heart attack in order to contribute to the design of effective secondary prevention services.	25 participants (34–80 years of age), 52% male.	Three important issues: whether MI is understood as an acute or chronic condition, whether the event is seen as mild or severe and the effect of advice that is at odds with patients’ experiences.
	Interview study with grounded theory approach.	Recruited from a larger RCT study sample. All post AMI and interviewed at 2 weeks and 5 months after hospital discharge.	These provide a rationale for the apparent low motivation for long-term lifestyle changes. However there are changes over time.

**Figure 2:**
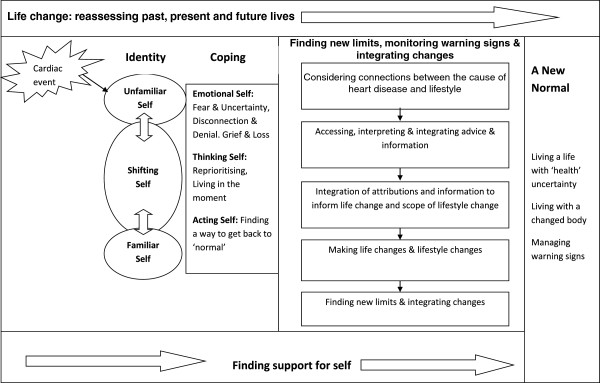
Synthesis of Lifestyle Change Process.

In line with Noblit and Hare’s methodology [20], Figure 2 presents a synthesis, comprised of second order (Themes and ideas generated in the results or discussion by researchers in each individual study) and third order constructs (Our collective understanding and interpretation of first and second order constructs reported in primary studies). The process of making lifestyle changes after a cardiac event was characterised by three second order constructs (‘Finding new limits and a life worth living’ , ‘Finding support for self’ and ‘A new normal’ and one third order construct (Life change: reassessing the past, present and future). Each construct was derived from those studies indicated in parentheses in Table 1.

### Life change: reassessing past, present and future lives

The threat to health posed by a cardiac event preceded a dynamic process in which participants reassessed their past, present and future lives in a cyclical process typically leading to significant life changes, not necessarily life style changes. As part of this transition participants experienced a change in their sense of identity. We conceptualise these different self-identities as ‘unfamiliar’ , ‘shifting’ and ‘familiar’ , together with dimensions of coping (emotional, thinking and doing). In looking across studies we noted evidence of different personal identities and coping responses depended upon the timing of the interviews.

In the first few days all participants experienced emotions of fear, uncertainty and disconnection; such responses were particularly marked amongst women (2, 3, 8, 9, 15, 17) and reflected the transition, from what we conceptualised, to be a ‘familiar’ , to an ‘unfamiliar’ self-identity. These emotions persisted in early weeks whilst participants confronted the change in their health which often involved thinking about their own mortality. The sudden health threat meant that, for many, this was the first time that they had faced their own mortality which was understandably confronting (21, 15). The loss of a ‘familiar’ sense of self represented a loss which was often accompanied by feelings of grief, and disconnection which linked to the notion of a shifting self-identity (12).

The change in life situation stimulated changes in emotions, thought processes and day-to-day activities. As part of this ‘thinking’ phase individuals typically made decisions about which aspects of their ‘life’ situation to change in response to their changed health status. Managing lifestyle change to improve or maintain their heart health was but one small part of responding to a changed health status and was not always a priority. Attention was often focused upon ‘bigger’ life decisions and considerations such as the potential threat to survival, continuation of work (paid or unpaid work in the home) and other life roles. This thinking, often in conjunction with wider family discussion, preceded significant life events such as retirement. As time progressed fear and uncertainty often receded. The majority of participants, but not all, made the transition to a new ‘familiar’ self. This transition process necessitated an acknowledgement, or acceptance of a changed health status. This often meant learning to live with uncertainty about future health, leading some participants to focus more upon the present than the future (9, 11).

‘It’s a scare and it’s a wake-up call. Hey, you’re older than you think you are, probably, and a lot more can go wrong than you think. Look how fast this happened. You can’t, like there’s a lot of things that I’ve been going to do over the years, like different albums and writing a little history about the family and things like that. Now I’m thinking I better get down to business and do this right away’ Sutherland and Jensen 2000 pg 671-2.

It seemed that this transition was a necessary part of progressing from an unfamiliar self-identity, through a shifting self, to a familiar self.

### Finding new limits and a life worth living

#### Considering connections between the cause of heart disease and lifestyle

During early recovery, participants typically pieced together preceding events to help them to ‘make sense’ of what had happened. An important aspect of this involved considering why they had developed heart disease. Most participants made the link between their lifestyle habits and the development of their heart disease (2,6,14). For others the connection was less clear as they perceived that they lived a ‘healthy life’ or had none of the conventional risk factors, producing a feeling of uncertainty (21). Linking lifestyle with their CHD seemed to be an important step in taking responsibility for health and was particularly marked amongst cigarette smokers (2,21). Although other unhealthy lifestyle habits were identified as contributory, such as lack of exercise and poor diet, these were often seen as secondary to the negative effects of stress (2,6,8,14,6).

*‘It’s one hundred percent stress. No doubt in my mind. Because my mind is like a computer. Its working, working, working. That’s why I don’t sleep at night. It’s going over and over and if anyone’s got a problem, I’ll worry about their problem as much as mine’* (MacInnes 2006, p113).

Participants’ understanding of their condition and its significance was not always logical. For example, some participants saw their condition as short term (14,26) but did not believe that there was a cure (14). Although many of the studies included in the synthesis stated that the identification of causal factors for CHD influenced the decision to make lifestyle changes, this does not reflect the complexity found within participant accounts. Some participants who identified unhealthy lifestyles as a causal factor during early recovery changed their views as time passed and decided not to tackle unhealthy habits. It is unclear whether the self-management of their lifestyle had changed because they had relapsed into former unhealthy habits, or because their perceptions about cause had altered to fit with their preferred lifestyle (25). An alternative explanation may be that participants required further interventions for angina symptoms which would challenge their beliefs about the value of lifestyle in making a significant impact upon their health status.

#### Accessing, interpreting and integrating advice and information

Participants and their families developed their own interpretation of preceding events and how these had impacted on their life. Participants wanted information about their condition and what they could do to prevent recurrence. Health professionals were a key source of information but a recurring theme was that advice was not sufficiently individualised and that participants needed someone to consider their life holistically to provide recommendations relevant to their personal situation (4,6,11,24).

*‘It says washing, shaving, dressing, reading, washing dishes and preparing light meals, playing cards, walking 2 to 4 miles per hour, climbing 1 to 2 flights of stairs slowly and shuffle board. It gives you an idea but I think it was meant for a 70 year old man. Not really relevant for me’* (Doiron-Maillet and Meagher-Stewart 2003. P17).

The amount of information provided was often overwhelming and in many cases perceived as contradictory (6,7,8,9,24).

*‘I was being told different things by different people. Much of it didn’t apply to me really. I found the whole experience confusing’* Gambling 2003 p72.

This contributed to feelings of uncertainty, confusion and frustration which impacted on quality of life (6, 7, 8, 9, 24).

*‘Not knowing the answers to these questions and not knowing how to ask for help, my natural tendency was to live to much less than my full potential’.* (Gambling 2003, p71).

As a result some participants rejected the information and advice given and described doing things by instinct [6].

Whilst health professionals viewed the advice they provided as key this view was not always shared by participants and their families (24). Information and advice from friends or family members who had been through a similar experience were considered as ‘truth learned from others’ and an important source of information (4) which provided a context for their own experience. This was a way of making sense through the experience of others (21, 22).

#### Making life changes & considering lifestyle changes

The initial motivation for making lifestyle change was often fear of disease progression, having a second AMI, disability or death.

‘Now I am faced with making all the changes. I have no choice now. Everything must change. I am so afraid to even put a French fry in my mouth. They said there are things you can change and things you can’t change. I need to put my energies into things I can change’ (Doirron-Maillet and Meagher-Stewart 2003, p21).

Feelings of gratitude to have survived motivated some participants to make the most of each day and tackle lifestyle changes that might increase their life span (2, 6-9, 15, 17-19, 26). Making lifestyle changes was often seen as a way of regaining some control over their disease, and regaining a sense of self (4, 9, 12, 13, 15-16, 18, 20-21, 24). Others felt unable to influence their disease and its progression which led to feelings of helplessness and hopelessness, which in turn reduced motivation to change lifestyle (6, 7-9, 11-12, 14, 26-27). Participants tended to focus upon the one lifestyle habit that they considered to be most important. Making too many changes at once was considered to be unmanageable and participants talked about the need for ‘realistic change’ (2,6,9,13,20). Dietary change was particularly challenging (6, 7-9, 16, 25). Some people built in “treats” as a way of balancing quality of life with the need to maintain a healthy diet (7-9, 13). Others talked of a “trade-off” in which they cut out one unhealthy food item but continued with others.

‘You incorporate and eliminate things in your diet until you get something you’re happy with, and you stick to it whether it’s good or bad’ (Gambling 2003, p73).

Participants focused upon what they could not have and what they needed to cut out (25). There was little mention of adding things in that could benefit their health and there was a general lack of clarity about what constituted a healthy diet, leaving participants feeling frustrated, confused and ultimately de-motivated (6-9, 19, 24). The concept of making ‘trade-offs’ was also evident amongst smokers who were trying to quit. They often chose not to make dietary changes as food was used to ward off nicotine cravings (6).

Most people with CHD appeared to have had led rather sedentary lifestyles prior to their cardiac event (6-8, 16). A lack of time due to work and other commitments was a barrier to regular exercise (5-8, 16), as were boredom with ongoing exercise regimes (7-8) and other physical conditions such as arthritis or back problems (9, 18-19).

Although stress was identified by many as linked to their CHD, participants were vague about what they could do to alleviate stress. Some chose to ‘ignore it’ or just to ‘live with it’ (2), whilst others avoided any stressful situations and took ‘life easy’ (20). Some made a conscious decision not to worry (3, 14, 20, 23) and others attempted to put stressful situations into the context of their survival and quality of life, so were more accepting of stressful events (20). Practical coping strategies included relaxation, yoga and exercise and some people changed their work and family life to decrease demands (20). Some participants reported feelings of scepticism about the value of lifestyle change, which increased over time, particularly if their disease worsened (6,9,17,19,27). The lack of a visible cue to indicate and measure the positive benefits of lifestyle change seemed to reduce motivation. In one study successful weight loss was regarded particularly positively, perhaps because it was a concrete and visible measure of success (3).

#### Finding new limits, monitoring warning signs and integrating changes

Activities that were previously part of daily life became challenging during early recovery, as participants learnt how to manage an ‘unfamiliar’ body. Their heightened awareness of physical symptoms led them to monitor their body more closely than before (4,10,11). This fuelled feelings of fear, uncertainty, vulnerability at the loss of the familiar physical self (11,15,19). Participants needed to establish new boundaries to their physical abilities (8). Some limited their physical activity for fear of ‘over-doing it’ (8) whilst others felt that the new boundaries could only be found by ‘going over them’ (8, 21). The fear of overdoing things often meant that people were too scared to exercise in case they caused another AMI (5-7, 24).

‘I think to encourage people to go back to exercise, rather than telling people that you are supposed to go back to exercise, but they get frightened so they don’t go back to it. I knew somebody who had a heart attack, and he flatly refused to go anywhere without the car, because he said he didn’t want to overdo it. But then he was bone idle before, so I suppose… he just carried on as before!’ (Gregory, Backett-Milburn et al. 2005, p17).

In contrast others pushed themselves too far, in order to ‘get back to normal’ as quickly as possible (2,9). This over-exertion could put a small number of participants, typically male, at risk. Ongoing physical symptoms were a source of concern as a possible marker of another cardiac event (3,19). For people with angina the pain was described as a ‘physical strait-jacket’ (15). As time elapsed participants typically became more confident about the self-management of their day-to-day activities of living which contributed towards a more ‘familiar’ self-identity.

‘At the beginning you don’t know what to do. You don’t know how far you should go on your own. And, then, after a while you need less help, and basically you don’t want any more help. You feel more freedom without knowing it. You know you are okay. Your limits broaden out, and you feel more comfortable with yourself.’ (Johnson and Morse 1998, p134).

### Finding support for self

The emotional impact of a cardiac event was significant and participants wanted reassurance and support from health professionals both in the immediate and long term (4, 7-8, 12-13, 19-20, 24). Family and friends also played a significant role in recovery and often supported and enabled participants to make lifestyle changes by joining them in new exercise regimes (7-8), assisting with dietary changes (1, 9, 23) and providing much needed motivation and encouragement. Conversely, family members could be over-protective which led to frustration and tension within the family, particularly for male participants (2, 10, 7-8, 12-13, 19). Some female participants felt over-protected (4, 21) but most wanted additional help and support (18). In some instances there was a reported lack of family support in making dietary changes (6, 9, 12, 23) and frustration voiced by participants as family members continued to smoke making quit attempts difficult (9, 23).

In some cases spiritual beliefs supported participants in coming to terms with their changed health status (9, 16, 23);

‘I have studied a lot of Hinduism before and I strongly believe that God is helping me through hard times’ (Mohan, Wilkes et al. 2006, p118).

Many participants expressed the need for more peer support, through buddy systems or support groups to help provide an essential understanding (4, 7-9, 18, 21);

‘….We are all in the same boat and that helps. It is just seeing people in the same boat, maybe not necessarily to the same extent, but we are all there in the same boat..it is much more meaningful coming from someone who knows just what it is like – someone you can relate to.’ (Doirron-Maillet and Meagher-Stewart 2003, p21).

Hearing about experiences from other people who shared the same condition made the information more meaningful.

### A new normal

Participants described the adjustment process as getting ‘settled’ , back to ‘normal’ , finding ‘a balance’ or simply as ‘getting on with life’ (4,11,13,21,26) including those who acknowledged that their heart disease was irreversible.

‘I want to get back to work, get back to normal, and get on with my life’ Condon and McCarthy 2006 p42.

Interestingly, the meaning of normal varied widely between individuals. What was occurring was a process of either adaptation or maladaptation that moved the individual to a ‘new normal’. For some people making lifestyle changes led to increased feelings of confidence which helped to maintain them. Over the longer term, lifestyle changes became established within daily routines and this ‘new normal’ reflected their altered health status and self-identity as well as a ‘life worth living’ (11, 15). Those who were less successful in making, and/or maintaining, lifestyle changes often felt powerless to influence disease progression. This tended to lead to an increase in feelings of fear, uncertainty about possible AMI recurrence and death (6, 9, 11, 14, 26-27) which accompanied an ‘unfamiliar’ or ‘shifting’ self-identity. These participants were less likely to make healthy lifestyle changes and progress to a ‘new normal’ characterised by the maintenance of the lifestyle habits that they perceived as pleasurable. In reality the process of making lifestyle changes could be considered to be ‘work’ that spans through a lifetime that takes place within the context of other significant ‘life’ changes such as retirement.

The process of making lifestyle change involved an iterative reassessment process in which individuals considered the adjustments they had made, or planned to make, and how these changes might impact upon their lives and whether they were realistic within the context of their own unique situation (3, 6, 9, 13, 18, 20-21). Evidence of this came from participants’ accounts of early recovery as they reported being unable to change everything at once and subsequently adjusted their personal goals to those that were considered more realistic. Some participants chose not to attempt to make lifestyle changes, partly because health professionals’ advice did not match their own experience or beliefs about their lifestyle or the perceived cause of their CHD. The fear of having another AMI may have provoked a level of fear too great to enable them to exercise or resume their familiar roles within family, social or work environments (3-4, 6, 7-11, 12, 14-15, 19). Overprotection by family members may have reinforced this ‘sick role’ thereby preventing change. The ‘new normal’ for these participants was one where they were further incapacitated and dependent on others which reduced their overall quality of life. Changes in self-identity occurred simultaneously with the transition to a ‘new’ normal.

Beliefs about the inevitability of disease progression, or a belief that CHD was inherited also led some participants to feeling powerless in influencing the course of their disease leading to minimal attempts at lifestyle change (6, 9, 11-12, 14, 26-27). The ‘new normal’ for these participants was likely to be functionally limited or resemble their old life, with negative impacts on future health.

There was some evidence that as participants began to feel physically well after AMI that their anxiety about their heart health diminished especially if they experienced no further physical symptoms. For some participants the reduction in fear led to reduced motivation to make lifestyle change, with subsequent reversion to old lifestyle habits (6, 12, 21). Some participants were able to either deny or distance themselves from their CHD diagnosis. It was possible that those who did not wish to make lifestyle changes would at some stage re-visit their decision. People often moved in and out of the adaptation process, and changes in life circumstances and health influenced this. Participants’ dialogues were frequently characterised by uncertainty and were at times contradictory, on the one hand, sharing their anxiety about their health, and on the other, explaining that nothing had changed and that they were back to normal. This appeared to be a reassurance mechanism that enabled the participant to locate some sort of emotional safety in the face of health uncertainty. There was a need at times to find some distance from the disease but also to acknowledge the situation and accept uncertainty. This was not possible for all participants.

‘I just feel the same [as I did before the heart attack] … obviously I am back to doing everything now that I’ve always done … [but] I suppose right in the back of your mind you do realise that once you’ve had one, well anyone can have one I know, but once you’ve had one you could have another one’ (Wiles and Kinmonth 2001, p165).

On-going physical symptoms, the taking of medication and the need for lifestyle change represented a constant and often unwelcome reminder of CHD for some participants.

‘I think probably the most difficult change has been getting accustomed to taking drugs every day. I find that a constant reminder, if you like. I find that difficult sometimes. It’s the fact that I am taking them. It is a constant reminder that I have had a heart attack, that I am not a fully fit individual. You think that if I don’t take these, if I just throw them all away, am I going to drop dead? I think about some things like that, now and again’. (Gregory, Backett-Milburn et al. 2005, p25).

The concept of acceptance came through in participants’ accounts; some appeared to face their illness ‘head on’ along with potential limitations, others struggled in and out of ‘acceptance’ , whilst others preferred to distance themselves from their cardiac event and “put it behind them”. The ambiguity and contradiction evident in some participants’ accounts suggested that acceptance was not easily attained. For some participants acceptance was possible by not allowing the CHD to be the prime focus of their lives and to let other aspects of their life take precedence (9, 11-13, 15, 18, 20-21, 23).

## Discussion

The aim of our synthesis (Figure 2) was to provide a person centred interpretation of the process of lifestyle change after a cardiac event grounded in the findings of 27 studies reporting on the experiences of over five hundred participants. In summary a cardiac event represents a significant life event which propels participants into an illness experience characterised by a changed self-identity. The rather sudden change in health status was shocking for participants and prompted them to assess their past, present and future lives as the ‘familiar’ self was lost and replaced by an ‘unfamiliar’ self. The distress that participants experienced in response to the loss of a familiar self-identity echoes the biographical disruption described by Bury [57] and the loss of ‘self’ reported by Charmaz [58]. Participants in our synthesis experienced uncertainty on two levels. The first was uncertainty about how to view their changed self-identity and the second linked to uncertainty about future health. Participants often mentioned the need to ‘get back to normal’; this process involved readjustment or recalibration; a process in which an individual shifted from an ‘unfamiliar’ self to a ‘familiar’ self. Some participants did not progress to a ‘new normal’ but became ‘stuck’ in an ‘unfamiliar’ self-identity with accompanying negative mood states. All participants experienced a sense of loss linked to the change in their health status. The five stages of the grief cycle (Denial, Anger, Bargaining, Depression and Acceptance) as described by Kubler-Ross (1969) [59] were evident in some participants’ accounts of their illness experiences. Those participants who became ‘stuck’ in an ‘unfamiliar’ self-identity tended to deny, or acknowledge, rather than accept, the change in their health status and displayed emotions that aligned with the denial and anger stages of the grief cycle [59].

This key finding emphasizes the importance of individualized psychosocial support during the adjustment that follows a cardiac event. The social and psychological elements of cardiac rehabilitation are recognised as important in promoting recovery but there is scant detail about how this support can be delivered in a busy clinical practice setting.

Empowerment has been mentioned as a key concept in a number of studies linked to self-management; Aujoulat et al. [60] wrote about the process of patient empowerment in people living with NCD’s and findings from her interview study lend support to our synthesis. She conceptualised the process of patient empowerment as a process of reconstruction that occurred in two parts; a restructuring of self and a restructuring of the illness experience [60]. Although participants in her study were diagnosed with NCD’s, other than CHD, there are several important and interesting parallels. In our synthesis the restructuring of self after a cardiac event was represented by the ‘shifting’ self-identity that occurred along with emotional and cognitive changes that influenced changes in day-to-day activities. The restructuring of the illness experience, which we have conceptualised as ‘Finding new limits and a life worth living’ , took place simultaneously and included the management of lifestyle change and medicines.

The restructuring of the illness experience was difficult for participants to process in part because of the lack of visible markers of CVD and concrete indications that lifestyle change was leading to an improvement in health. Lifestyle changes that could be measured such as cholesterol concentration provided concrete evidence of the benefit of making lifestyle changes. As part of the restructuring of the illness experience the majority of participants wanted relevant health information. This knowledge and information seeking behaviour is recognised as an important aspect of empowerment because it enables people to separate the disease from their sense of self [60-62].

All participants tried to make links between their prior lifestyle habits and the onset of their heart disease but not all were able to make sense of how, or why, their heart disease had developed. They integrated health information into their own unique illness model which often, but not always, influenced the way in which they managed their lifestyle and medicines. Many participants also tested boundaries linked to levels of physical activities and day-to-day activities to establish how much they could safely do. The ‘testing’ shared some features of the bargaining stage of the grief process [59]. Secondary prevention initiatives, such as taking medications or attendance at cardiac rehabilitation, represented a potent and often unwelcome reminder, for some participants, of their own mortality. Such cues served to influence participants’ level of engagement and adherence with lifestyle change and medicine management and could trigger a move back into a ‘shifting’ self-identity. Making significant life changes, such as early retirement, was a common theme across the adaptation process. Lifestyle change and medicines management did not always take centre stage in life, but were rather nested within a larger and more encompassing life change process. That is not to say participants did not recognise the importance of lifestyle change and medicines management but other priorities often linked to their ‘being’ took precedence. The way that participants chose to adopt some lifestyle changes, and ignore others, was reminiscent of the bargaining stage of the grief cycle [59]. Accessing support from family friends and health professionals was an important adjunct to recovery and participants experienced different levels of support which could be either enabling or constraining.

A ‘new normal’ was found which was characterised by an awareness of some future health uncertainty. Although participants talked about ‘getting back to normal’ , this was a label rather than a reality, as most participants realised that there had been a fundamental change which required them to live day-to-day life in a different way and with some uncertainty about their future health. This often led to the monitoring of physical sensations which could be interpreted as warning signs or ignored depending upon the individual.

From the participant’s perspective lifestyle change was important as an approach to prevent disease recurrence but was nested within a much larger ‘life change’ process that reflected movement between an ‘unfamiliar self’ and ‘familiar self’ driven by a powerful urge to ‘get back to normal’. The precise nature of what was ‘normal’ existed on a continuum of change stimulated by external factors.

To the best of our knowledge this is the first qualitative synthesis, which has used meta-ethnography, to present a person centred model of the lifestyle change process which is contextualised within the wider illness experience of living with CHD. We believe that the synthesis is a valuable addition to the literature because it collates and crystallises insights from a large and rather nebulous body of literature around the more focused issue of behavioural change, something that individual studies on the same subject have not achieved. However whilst this study is original and innovative there are some limitations that should be acknowledged.

Meta-ethnography is a somewhat controversial methodology mainly because of a lack of agreement about some aspects of the analytical process. For example there is no consensus about sampling approaches and the value of combining qualitative studies using diverse methodologies and analytical processes is contentious. To militate against this we carefully considered potential sources of heterogeneity across studies throughout the synthesis process. The transferability of findings is an important consideration; the themes that informed the synthesis reflect the experiences of individuals living in 8 countries which may not necessarily be transferable across all cultural settings. Moreover the synthesis is the result of concepts that were of interest to, and reported by, the participating researchers. For that reason this unique interpretation may not necessarily represent the entire range of relevant concepts.

Regardless of such methodological limitations there is unanimous agreement about the importance of better understanding patients’ perspectives about making lifestyle change to reduce coronary risk. This knowledge is a valuable adjunct that serves to complement existing quantitative research identifying effective interventions to support people diagnosed with CHD to make healthy lifestyle changes; the only non-pharmacological approach known to slow CVD progression.

Findings have a number of implications with regard to clinical practice. Lifestyle modification programmes, such as cardiac rehabilitation, are effective in supporting behavior change compared to routine care [63] but provision of such programmes is variable across Europe. Current services tend to focus upon interventions to support physical activity and exercise delivered 6-8 weeks after discharge from hospital. Whilst this approach is understandable, from the perspective of minimising risk, this pattern of delivery does not provide the necessary psychosocial support. The early discharge period is particularly anxiety provoking and support should ideally be provided immediately after discharge. One of the key challenges is the provision of individualised care as each individual has a different experience and not all are at a stage where they wish to fully engage with making lifestyle change. For those patients that are ready to make changes it is important to provide self-management support as they ‘learn’ about their new boundaries which may, or may not, reflect the priorities of the attendant health professional.

## Conclusions

The final synthesis presents an interpretation, not evident in the primary studies, of a person-centred model to explain how the self-management of lifestyle change is situated within much ‘wider’ life changes. The magnitude of an individual’s response to their changed health status varied. During early recovery self identity shifted and participants experienced some degree of distress. The emotional stages of grief were evident in the adjustment process that followed. This new finding highlights the need for health professionals to have an opportunity to learn about how to provide support for people experiencing grief; a subject not typically covered in current curricula. Whilst healthcare professionals recognise lifestyle change as a priority, this view is not necessarily shared by patients and their families which can be a potential source of tension. The level of engagement with lifestyle change reflects an individual’s unique view of the balance needed to manage ‘realistic change’ whilst leading to a life that is ‘worth living’. Findings accentuate the importance of providing person centred care that aligns with both the psychological and physical dimensions of recovery which are inextricably linked.

## Competing interests

The authors declare that they have no competing interests.

## Authors’ contributions

FA conceived the original idea for the study and led on the development of the funding bid that supported the work with input from SJC. All authors contributed to the development of the search strategy, data analysis and synthesis development. JH conducted the search with input from FA and SJC. FA led on manuscript development; all authors contributed to the draft, read and approved the final manuscript.

## Pre-publication history

The pre-publication history for this paper can be accessed here:

http://www.biomedcentral.com/1471-2261/14/96/prepub
